# Application of VHH-Immobilized Cryogel-Based Immunoaffinity Chromatography for Isolation of Extracellular Vesicles

**DOI:** 10.3390/molecules30224337

**Published:** 2025-11-08

**Authors:** Jovana Terzić, Lidija Filipović, Ninoslav Mitić, Sanja Stevanović, Jugoslav Krstić, Ario de Marco, Julie Courraud, Milica Popović

**Affiliations:** 1 Institute of Chemistry, Technology and Metallurgy, National Institute of the Republic of Serbia, University of Belgrade, 11000 Belgrade, Serbia; jterzic@chem.bg.ac.rs (J.T.); sanjas@ihtm.bg.ac.rs (S.S.); jugoslav.krstic@gmail.com (J.K.); 2 Innovative Centre of the Faculty of Chemistry, 11158 Belgrade, Serbia; filipoviclidija29@gmail.com; 3 Institute for the Application of Nuclear Energy (INEP), University of Belgrade, 11080 Belgrade, Serbia; ninoslavm@inep.co.rs; 4 Laboratory for Environmental and Life Sciences, University of Nova Gorica, 5000 Nova Gorica, Slovenia; ario.demarco@ung.si; 5 Proteomics Core Facility, School of Science, National and Kapodistrian University of Athens, 15784 Athens, Greece; 6 Section of Clinical Therapeutics, Department of Medicine, School of Health Sciences, National and Kapodistrian University of Athens, 15771 Athens, Greece; 7 Faculty of Chemistry, University of Belgrade, 11158 Belgrade, Serbia

**Keywords:** extracellular vesicles, cryogels, nanobodies, immunoaffinity chromatography

## Abstract

Extracellular vesicles (EVs) are nanosized structures involved in intercellular communication that have high potential as disease biomarkers and for the delivery of therapeutic cargos. However, translation to the clinic is hampered by time-consuming, low-yield, and poorly reproducible EV isolation methods. We describe a cryogel-based immunoaffinity chromatography system that exploits single-domain VHH antibodies as capture elements for the selective isolation of EVs from human plasma. Supermacroporous cryogels functionalized with five unique anti-EV VHHs (total immobilization capacity ~500 µg/g) were prepared, yielding a highly permeable and hydrophilic support. They were captured and eluted under mild conditions, and their morphology and identity were confirmed by SEM, AFM, NTA, and flow cytometry. Proteomic profiling of the isolated samples identified 234 proteins, of which 63% were ExoCarta-listed exosomal proteins; contaminants such as albumin and apolipoproteins were also identified. The purification method provided samples with ~2 × 10^9^ EVs/mL, with EV median size of 135 nm and consistent protein-to-lipid ratio across three independent isolations (CV < 10%). This study demonstrates that VHH-functionalized cryogels (VHH-SMC) are a rapid and reproducible EV purification method that represents a promising alternative to conventional ultracentrifugation- or precipitation-based protocols. While optimization of nanobody density and reduction in plasma protein carryover are still necessary, the platform holds potential for scalable EV enrichment, a condition that can significantly speed up biomarker research and clinical diagnostics.

## 1. Introduction

Extracellular vesicles (EVs), heterogeneous membrane-enclosed particles released from cells, play important roles in both physiologic and pathologic processes [1]. Extracellular vesicles (EVs) are heterogeneous, membrane-bound particles released by cells and involved in intercellular communication. EVs derived from eukaryotic cells are commonly categorized into three major classes exosomes, microvesicles, and apoptotic bodies based on their cellular origin and biogenesis pathways. However, additional types of EVs have been described in other biological systems, including bacterial outer membrane vesicles (OMVs) and outer-inner membrane vesicles (OIMVs) [2]. The recently updated MISEV2023 guidelines provide comprehensive recommendations for EV terminology, classification, and characterization, promoting clarity and consistency across different biological contexts [3]. Exosomes are derived from multivesicular bodies (MVBs), whereas microvesicles are shed directly from the cell membrane, and apoptotic bodies originate from cells undergoing apoptosis. Exosomes are biologically active EVs with a bilayer membrane structure of approximately 30–150 nm in diameter that are secreted from living cells, while microvesicles have diameter between 50 and 1000 nm [4,5]. EVs carry biologically active substances contained in the original cells, such as nucleic acids, proteins, lipids, and metabolites [6,7]. The composition of EVs may vary significantly based on the pathophysiologic status of the parent cell [8]. It is becoming increasingly evident that the majority of EVs have specialized functions and are essential for processes such as intercellular signaling, cellular waste management, and coagulation [9]. EVs are promising candidates as clinical biomarkers in the diagnosis and monitoring of a variety of diseases due to their specific molecular cargo [6], contributing to the advancement of molecular diagnostics and personalized medicine. Due to their small size and heterogeneity, the detection and classification of vesicles still present major challenges. The main obstacle for practical applications of EVs is that the procedures commonly used for their separation from body fluids are cumbersome, hard to standardize, and often lead to inconsistent results [10,11]. Vesicles are commonly isolated using a combination of ultracentrifugation and size-exclusion chromatography, allowing for their separation based on size [12]. However, these methods are time-consuming, often associated with limited purity and questionable reproducibility, making inter-laboratory consistency difficult to ensure [13]. Furthermore, they do not discriminate between physiological and pathological EVs. Immunoaffinity chromatography offers an effective alternative for isolating EVs, and antibody fragments, such as single domain antibodies (VHH), represent inexpensive reagents to use for EV capture with respect to conventional IgGs [14,15]. VHHs used in this work have been obtained by panning a naïve library against EVs isolated from cell culture supernatant and have been applied for EV isolation starting from different biological sources [14,15,16].

**Figure 1:**
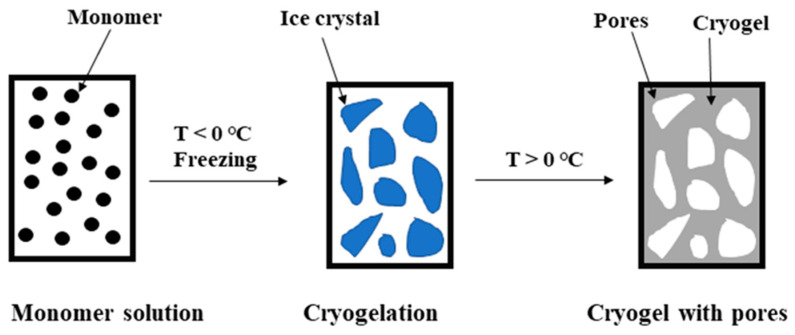
Schematic illustration of SMC synthesis.

Supermacroporous cryogels (SMC) can be prepared by free radical copolymerization of acrylamide, bis-acrylamide, and allyl glycidyl ether in an aqueous medium by crystallizing fluids and copolymerizing monomers under freezing conditions [17,18] and have been successfully applied as chromatographic materials and carriers for the immobilization of particles [19,20]. Water crystallization is crucial for SMC properties, as the formed ice crystals serve as templates during cryogel formation [21]. At temperatures below the solvent’s crystallization point, water freezes, while dissolved monomers and initiators concentrate in the unfrozen regions, where polymerization occurs. In this way, the transition of the solvent from the liquid to the solid phase occurs, enabling the formation of a highly porous SMC structure. Upon thawing the gel to room temperature, the ice crystals melt, leaving behind macropores (Figure 1) [22,23,24]. SMC supermacroporosity enables the separation and purification of components from complex samples such as biological fluids [18]. Hydrophilic monolithic cryogels have an elastic and spongy structure hosting interconnected macropores with a diameter up to 100 µm [25,26]. This highly permeable substrate provides very little resistance to flow through [27] and offers an excellent solid support for the attachment of VHHs necessary for the isolation of supramolecular structures such as EVs. In this work, we have, for the first time, set up a VHH-based immunoaffinity SMC chromatography system for the isolation of EVs from the plasma of healthy donors.

## 2. Results

### 2.1. Characterization of SMC

**Figure 2:**
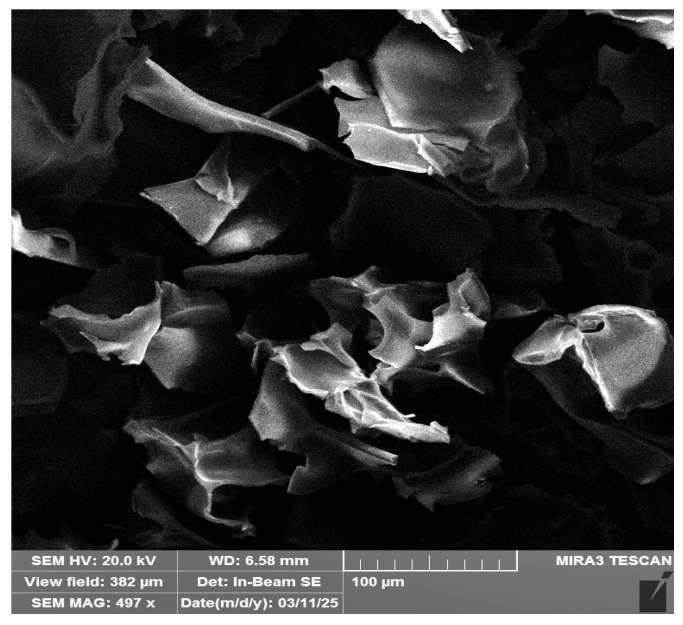
SEM images of SMC.

The SMC composed of acrylamide (AAm), bis-acrylamide (MBA) and allylglycidyl ether (AGE) was successfully synthesized and afterward characterized by measuring its swelling behavior, flow rate, and hydraulic permeability. The physicochemical characteristics of the SMC are summarized in Table 1. SMC is highly porous with determined modal pore diameter of 20–100 µm. SMC has characteristic internal structure of cryogels with heterogeneous interconnected pores with thin and smooth walls (Figure 2). The mercury intrusion porosimetry results for dried SMC before and after VHH functionalization are presented in Table 2. (Supplementary Figure S2, integral and differential pore size distribution curves). Parameters such as total intruded Hg volume (Vtot), bulk density, average pore diameter (Davg), and most frequent pore diameter (Dmax) confirm the successful synthesis of highly porous SMC, even after conventional drying (48 h in a laboratory oven at 60 °C). A porosity of nearly 80%, combined with a monodisperse pore size distribution exhibiting a maximum at approximately 29.5 μm, and 90% of the total volume of intruded mercury (V0.9) occurring in pores larger than 25 μm, reveals the macroporous nature of the synthesized material. This reflects a pore structure concentrated within a narrow range of several tens of micrometers. Furthermore, the strong agreement between porosity values derived from true density (He pycnometry) and apparent density (Hg porosimetry)—80.3% and 79.7%, respectively—suggests that pore content smaller than the MIP detection limit is practically negligible. The SMC modification with VHH resulted in a notable change of over 12% in true density, indicating skeletal structure alteration due to successful VHH binding to the initial SMC through the applied modification process. The functionalization also yielded somewhat unexpected changes: Vtot increased nearly 2.2-fold, bulk density decreased by a simultaneous 48% in both Davg and Dmax shifted toward larger pore diameters. The pore diameter range accounting for 90% of intruded volume narrowed and shifted toward larger sizes (45–60 μm). The resulting porosity exceeded 90%, making the material comparable to freeze-dried cryogels, although freeze-drying was not used for VHH-SMC preparation.

### 2.2. Isolation of EVs on SMC

**Figure 3:**
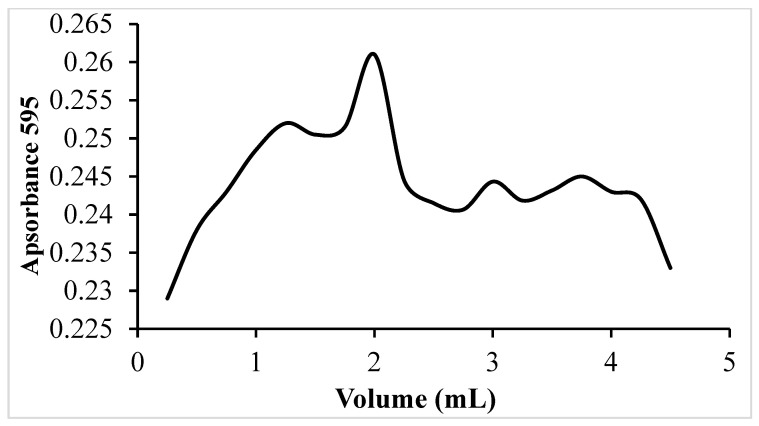
Elution profile of EVs isolated from human serum by VHH-functionalized SMC. Elution was monitored by measuring the absorbance at 595 nm.

The SMC was functionalized with a total of 500 μg of VHH antibody per gram of SMC by adding 100 μg of each of the five different VHHs. The prepared cryogel was used to isolate EVs from the human plasma of healthy donors. Plasma samples (500 µL) were diluted with 4 mL of 0.1 M sodium phosphate buffer (pH 7.4) and the resulting 4.5 mL of sample were applied to the SMC column using a peristaltic pump. After washing the unbound components, the bound vesicles were successfully eluted lowering the pH. Individual 250 µL fractions were collected and their absorbance at 595 nm was used for assessing the elution profile in order to determine the fractions enriched in vesicles. The elution profile (Figure 3) shows multiple peaks in the early fractions, followed by a gradual signal in the subsequent fractions. Protein concentration measured by BCA assay indicated values around 10 µg/mL.

### 2.3. Flow Cytometry Analysis

**Figure 4:**
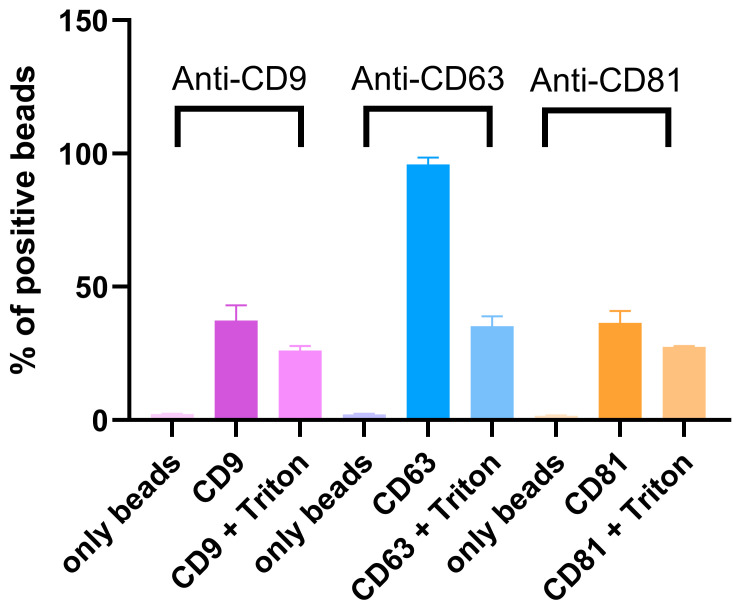
Analysis of the membrane biomarkers of EVs isolated with using SMC-VHH immune-affinity matrix. Three common EV surface markers were targeted with specific antibodies: Anti-CD63-AlexaFluor488, Anti-CD9-PE, and Anti-CD81 PE/Dazzle. Triton X-100 controls were used for confirming vesicular structure. Error bars represent standard deviation of triplicates.

Assessment of tetraspanin markers in the eluted samples was performed using flow cytometry. A pronounced fluorescence shift for all three extracellular EV markers: CD63, CD81, and CD9 was observed (Figure 4). The addition of Triton X-100, a non-ionic detergent that disrupts lipid membranes, resulted in a reduction in fluorescence intensity, indicating that the fluorescent signal was associated with intact vesicular structures.

### 2.4. Number and Morphology of Isolated EVs

**Figure 5:**
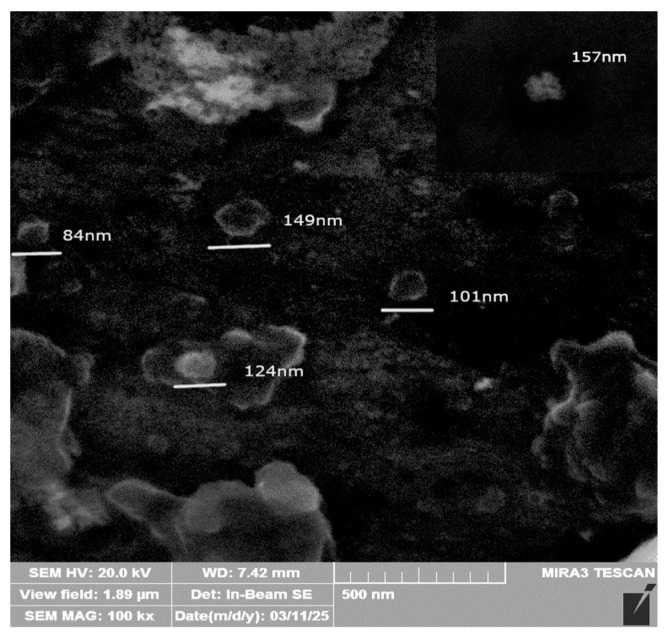
Scanning electron image (SEM) of extracellular vesicles.

**Figure 6:**
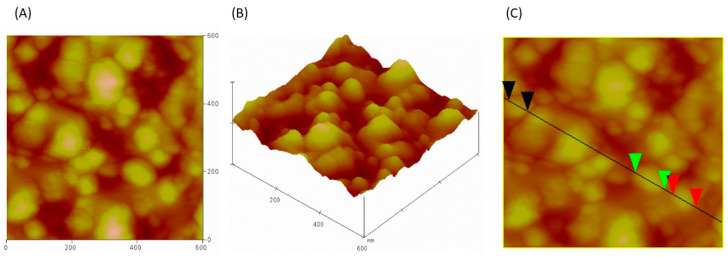
AFM analysis of isolated EVs: (**A**) 2D image, (**B**) 3D surface rendering, (**C**) cross-sectional profile.

**Figure 7:**
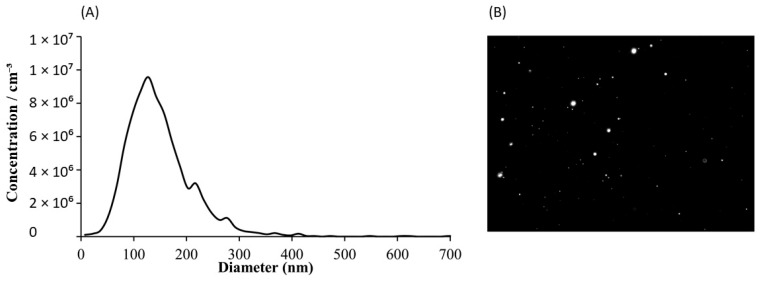
Nanoparticle tracking analysis (NTA) of isolated extracellular vesicles. (**A**) Size distribution-particles ranged in size from 50 to 200 nm, with the majority falling between 100 and 150 nm. (**B**) Representative video frame capture. Data on size distribution represent the mean values of three independent measurements.

The SEM analysis (Figure 5) demonstrated that the particles exhibited spherical morphology, with well-defined structures consistent with EV morphological characteristics. AFM analysis illustrate representative 2D and 3D pictures, in which the vesicles exhibit a characteristic spherical morphology (Figure 6A,B). Their diameters were estimated based on the cross-sectional profile shown in panel C. The measured sizes ranged from 60 to 90 nm: the black marker corresponds to a vesicle measuring 62.7 nm, the green to 98.2 nm, and the red to 81.1 nm. NTA quantified the concentration (1.9 × 10^9^ particles/mL) and indicated that diameters ranged from 50 to 200 nm (Figure 7), and awith median vesicle diameter of 135.6 ± 1.19 nm. The EV features are summarized in Table 3.

### 2.5. Reproducibility of EVs Isolation

**Figure 8:**
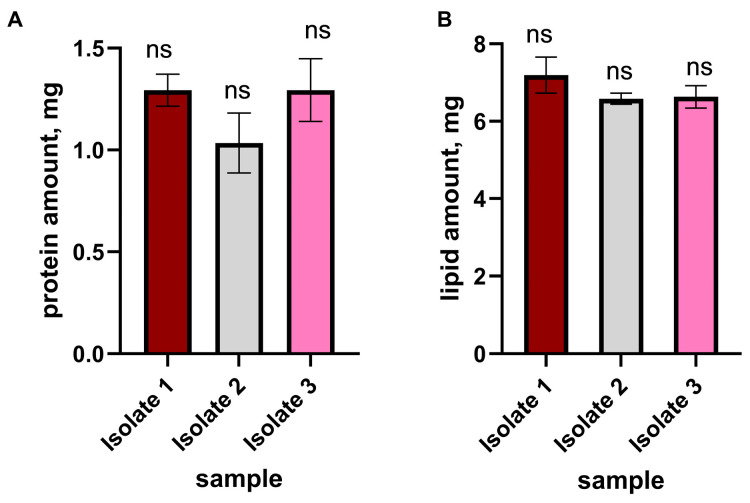
Reproducibility of EV isolation using VHH-SMC. Serum samples from healthy donors were subjected to three independent EV isolations. Isolates were analyzed for protein (**A**) and lipid (**B**) content. Data are presented as mean ± SD from technical triplicates (*n* = 3). A significance level of *p* ≤ 0.05 was used for analysis of variance, implemented using the one-way ANOVA test followed by the Tukey’s post hoc test (*p* ≤ 0.05). Values with a significance level of *p* ≤ 0.05 were considered statistically different. ns: not significant.

The reproducibility of the EV isolation using VHH-functionalized SMC was evaluated comparing the results from three independent experiments. For each isolation, protein (Figure 8A) and lipid (Figure 8B) contents were quantified in technical triplicates and are shown as mean ± SD. The measured L/P ratios were 5.559 ± 0.125, 6.365 ± 0.164, and 5.123 ± 0.163 for the three isolations, respectively (Table 4).

### 2.6. MS Analysis of EV Proteome

**Figure 9:**
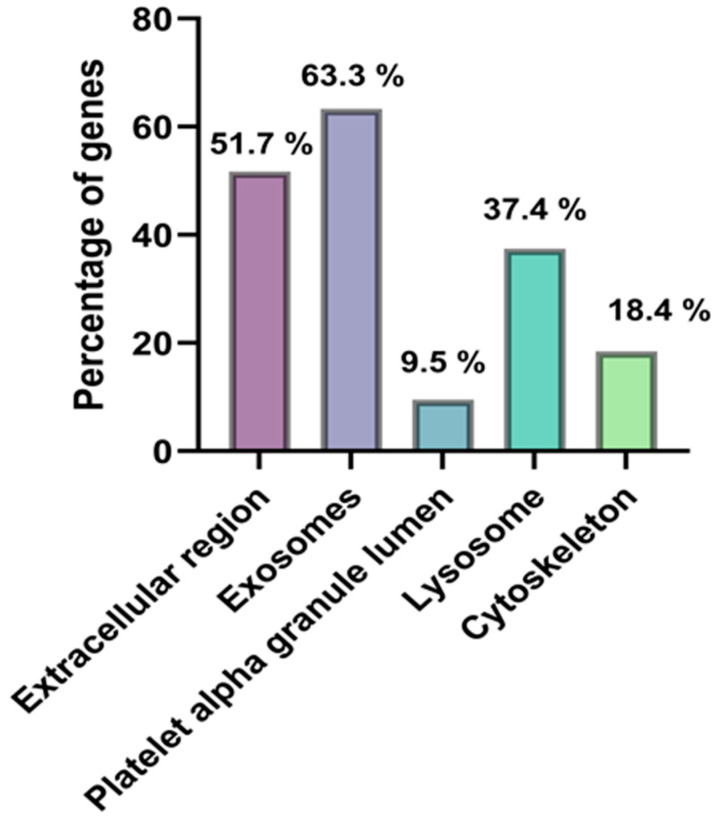
Functional enrichment analysis of proteins identified in serum-derived EVs using FunRich (Cellular Component category). The highest enrichment was observed for exosome (63.3%) and extracellular region (51.7%), with statistical significance (*p* = 0.05).

**Figure 10:**
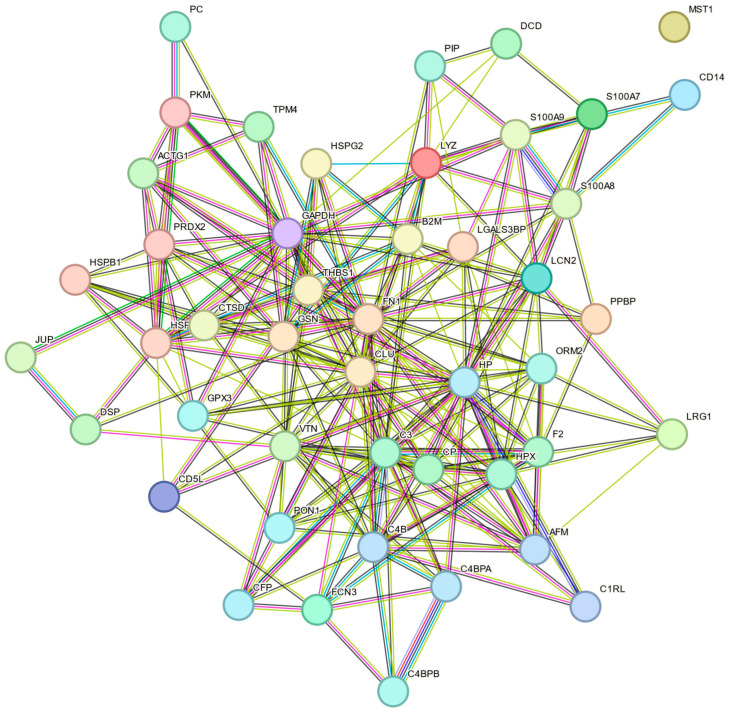
Protein–protein interaction network of EV proteins generated using the STRING database. Protein–protein interaction network was generated using the STRING database with a medium confidence score (0.4). Edges indicate known or predicted associations based on selected evidence channels: turquoise (curated databases), purple (experimental data), yellow (text mining), and dark blue (co-expression).

A total of 232 distinct proteins were identified in EVs isolated from plasma using mass spectrometry (Supplementary Table S1, proteins present in at least three replicates). A substantial portion of these proteins has been previously reported to be specific to EVs, as confirmed by cross-referencing with publicly available EV databases, including ExoCarta and Vesiclepedia. Table 5 lists representative EV-associated proteins grouped by functional categories. These include proteins involved in cell adhesion, immune signaling, and cargo transport, such as LGALS3BP, CD14, LYZ, FN1, HSPA5, and S100A9. In addition to EV-associated proteins, common contaminants were also detected, including abundant plasma proteins (e.g., ALB, APOE), keratins (e.g., KRT1, KRT9), hemoglobin, and immunoglobulin fragments. Their presence reflects co-isolation during EV recovery. The functional enrichment analysis of the identified proteins, carried out within the Cellular Component GO category using the FunRich tool, showed that the proteins in the highest percentage belonged to the exosome (63.3%) and extracellular region (51.7%) categories. Additional enrichment was also noted in categories such as lysosomes (37.4%), cytoplasmic cytoskeleton (18.4%), and platelet alpha-granule lumen (9.5%). The data obtained from this analysis are shown in Figure 9. A protein–protein interaction (PPI) network was constructed using the STRING database to investigate potential functional relationships among the EV-related proteins. The resulting network contains 46 nodes and 236 connections. STRING analysis revealed a highly significant enrichment of protein–protein interactions (PPI enrichment *p* < 1.0 × 10^−16^), confirming that the identified proteins are functionally connected rather than randomly associated. The visual representation of the network (Figure 10) shows that most of the identified EV-proteins form one connected component, with defined functional clusters. There is a cluster of proteins associated with innate immunity and the complement system, such as C3, C9, and HP, then a group of proteins involved in cell structure and intercellular adhesion (FN1, ACTG1), as well as a cluster of proteins involved in stress-response and redox regulation (HSPA8, PRDX2). Proteins involved in metal and lipid transport were also identified, including LCN2 and S100A8. Certain proteins, such as GAPDH, FN1, and HP, are associated with multiple clusters and function as hubs within the network.

## 3. Discussion

EVs are now recognized as essential in cell biology and communication, but were previously overlooked due to their long history of being misidentified as “cellular waste” [28,29]. A more significant investigation of these membrane-bound particles started in the early 2000s, resulting in accelerated scientific interest. In 2011, the term “extracellular vesicles” was coined to refer to lipid bilayer-containing particles released actively by cells. It is now well established that bacteria, archaea, and eukaryotes all secrete EVs [30,31]. Extensive research concentrated on the involvement of EVs in diseases like cancer and autoimmune disorders. Wider translation of EVs in the clinical settings has been hampered by ineffective and non-reproducible methods. For extracellular vesicles isolation, various techniques, such as ultracentrifugation, size exclusion column chromatography (SEC), and polymer precipitation, are used [32]. Ultracentrifugation has long been the standard method, but requires specialized equipment and can lead to contamination by non-EV particles and damage to the vesicles themselves due to high forces [33]. SEC enables size separation and preservation of EV integrity, but often does not provide sufficient purity because it does not remove all protein contaminants [34]. Polymeric precipitation is simple to apply but results in low specificity and a large amount of co-precipitated proteins [35]. On the other hand, affinity methods, especially immunoaffinity chromatography, allow highly specific isolation of EVs based on the expression of surface molecules [31]. Nanoantibodies derived from naïve libraries enable selective and stable isolation of specific EV subpopulations for research and diagnostics [15]. VHH can be fused to tags that simplify their biotechnological use and such constructs bind to markers on the surface of EVs, allowing their isolation from complex biological material [14].

Porous polymer monoliths are a novel class of materials that have emerged in the past two decades. These materials are produced using a straightforward molding process conducted within a closed mold [24]. Cryogelation techniques have been employed to create macroporous monolithic materials from hydrophilic monomers and polymers for biomedical applications [22,27]. Cryogelation allows for the creation of monolithic matrices that are elastic and mechanically stable, with large interconnected pores that are readily permeable to aqueous protein solutions and cell suspensions that otherwise would obstruct the conventional chromatographic columns manufactured using beads. Allyl glycidyl ether is commonly used to introduce epoxy groups for subsequent functionalization with bioactive agents [27].

This work describes the characteristics of an immunoaffinity material obtained by exploiting five anti-EV VHH nanoantibodies to functionalize monolithic SMC. This strategy enhances EVs capture capacity and the data collected so far indicated its consistency. Since monoclonal immunoaffinity-based capture can potentially isolate only specific subsets of vesicles that may not accurately represent the whole range of the original sample, we opted for the mix of five monoclonal VHH. The binding affinities of such VHH nanoantibodies are not yet available, but it is generally presumed that nanoantibodies recovered from naïve libraries have moderate affinity. This condition turns out to be optimal because the monolith surfaces represent a polyvalent binder element with elevated avidity effect that is effective for EV capture, but the moderate affinity of the single binders still allows the elution of intact vesicles under mild conditions. The characterization of SMC indicated that the material possesses structural and physical features that are beneficial for affinity chromatography and the isolation of biological particles. The high swelling ratio (16 g H_2_O/g gel) indicates significant hydrophilicity and the cryogel ability to retain considerable water within its structure, which is crucial for preserving the biological activities of attached biomolecules and preventing denaturation throughout the isolation process. The measured flow rate (8 mL/min) and the computed hydraulic permeability (1.296 × 10^−10^ m^2^) validate that the cryogel features an open and well-integrated macropore network. The SMC structure facilitates the unobstructed transit of molecules and vesicles across the matrix, with minimal flow resistance.

Mercury porosimetry analysis revealed a significant increase in total porosity from 80.3% to 90.8% alongside an expansion in average pore size following the functionalization sequence (EDA → GA → nanoantibodies). Unlike typical solid supports, where modification reduces porosity by pore filling, VHH-SMC showed a substantial increase—an effect that can be attributed to multiple factors. These include altered polymer-water interactions during functionalization, potential removal of labile matrix components, and possibly the formation of secondary VHH-based supermacroporous frameworks, effectively creating a new, more open porous architecture that uses the initial network as a scaffold. This interpretation is consistently supported by the distinct shift of Dmax toward larger diameters in both differential and integral PSD curves. This substantial enhancement can be attributed to several interconnected mechanisms. The introduction of polar amino groups during ethylenediamine treatment promotes increased swelling and hydration of the polymer network, while subsequent crosslinking with glutaraldehyde effectively “locks” the structure in this more expanded configuration. During nanoantibody immobilization and subsequent washing steps, partial removal of weakly associated matrix segments may occur, generating additional interchannel connections and further opening the structural framework. Comparable porosity increases following chemical functionalization have been documented in other hydrogel systems, including aminated lignin hydrogels and gelatin cryogels [36]. Furthermore, the potential contribution of VHH nanoantibody aggregates forming secondary supermacroporous structures accessible to mercury intrusion cannot be excluded.

Orthogonal analytical approaches confirmed the successful isolation of EVs from plasma. Flow cytometric analysis indicated a clear fluorescence shift in EV samples incubated with markers CD9, CD63, and CD81. Treatment with Triton X-100 caused a sudden signal loss, supporting the observation that fluorescence required intact membrane vesicles rather than protein aggregates or other contamination. Nanoparticle Tracking Analysis (NTA) indicated that both particle concentration and median size were within the expected values for serum-derived EVs. In our study, the applied isolation protocol yielded approximately 3.8 × 10^9^ particles per mL of plasma, as determined by NTA. This value is consistent with previously reported ranges for plasma-derived EV preparations obtained using size-exclusion chromatography or differential ultracentrifugation, which typically yield 10^9^–10^10^ particles/mL, depending on pre-analytical conditions and measurement settings [14]. It should be acknowledged that NTA quantifies all nanoparticles within the analyzed size range, and therefore the reported values likely represent an upper estimate of the true EV concentration. Nevertheless, the obtained particle numbers are in good agreement with comparable methodologies reported in the literature. High-resolution microscopic methods (AFM and SEM) confirmed the spherical shape and homogeneity of the isolated particles, with diameters corresponding to the characteristic range of EVs. Proteomic analysis by mass spectrometry identified a total of 232 different proteins. A large part of them have already been previously recorded in the ExoCarta and Vesiclepedia databases, confirming that the isolated samples correspond to the EV-proteome. Among these, there were those involved in cell adhesion (FN1, LGALS3BP), immune processes and signaling (CD14, LYZ, S100A9), as well as proteins related to stress response (HSPA5, PRDX2). Taken as a whole, the integration of data obtained by flow cytometry, NTA, microscopy, and proteomics unequivocally confirms that the isolated particles are indeed EVs and that they possess the expected morphology and biochemical composition, as well as a representative protein profile.

The acrylamide-based cryogel enabled the rapid and straightforward isolation of highly pure EV samples, ensuring both speed and sample quality. However, the binding capacity and overall capture efficiency may be limited by the comparatively low density of immobilized nanoantibodies on the cryogel surface, which is estimated to be approximately 500 µg of protein per 1 mL of cryogel. Future optimization efforts could focus on improving the immobilization density or enhancing the orientation of nanoantibodies. Finally, we still need to optimize the conditions to minimize the EV sample contamination with soluble proteins highly represented in body fluids. Surface coating and more extensive washing will be tested, taking as models protocols developed for preparing ELISA or biosensor substrates.

## 4. Materials and Methods

### 4.1. Chemicals

Acrylamide (AAm, 99%, Sigma-Aldrich, Saint Louis, MO, USA), N′,N′-methylene-bis(acrylamide) (MBA, 99%, Sigma-Aldrich, Saint Louis, MO, USA), Allyl glycidil ether (AGE, 97%, Thermo scientific, Waltham, MA, USA), N,N,N′,N′-tetra-methyl-ethylenediamine (TEMED, 99%, Fishcer Scientific, Waltham, MA, USA), Amonium-persuflate (APS, 99.5%, Betahem, Belgrade, Serbia), Ethylendiamine (EDA, 99%, Thermo Scientific, Waltham, MA, USA), Glutaric dialdehyde (25% *w*/*v*, Thermo Scientific, Waltham, MA, USA), cell culture media and reagents, fetal bovine serum and bovine serum albumin (BSA) were from Sigma Aldrich (Steinheim, Germany). Anti-CD63 (clone TS63) antibody, anti-CD9 Phycoerythrin (PE)-labeled (clone MM2/57) antibody and Protein Quantification BCA Protein Assay Kit were from Abcam (Cambridge, UK). Alexa Fluor^®^ 488 anti-human CD63 (clone H5C6) antibody and PE/Dazzle™ 594 anti-human CD81 (TAPA-1) antibody (clone 5A6) were from Biolegend (San Diego, CA, USA). All other chemicals were p.a.

### 4.2. Production of Super Microporous Cryogel

Supermacroporous AAm-AGE cryogel was prepared according to the free radical cryo-copolymerization method combining solvent crystallization and polymerization of monomers. Allyl glycidyl ether is used to insert reactive epoxy groups. The monomers (0.780 g AAm, 0.130 g MBA, 365 µL AGE) were dissolved in deionized water, and the solution was stirred for 1 h to ensure complete dissolution and homogeneity of the monomer mixture. The total concentration of monomers was 6% (*w*/*v*). Radical polymerization of the reaction was initiated by TEMED (10 µL) and APS (14 mg) in an ice bath. The reaction mixture was transferred into a plastic syringe with a sealed bottom. The polymerization solution was frozen at −18 °C for 18 h and thawed at room temperature. The column was washed with deionized water.

### 4.3. Characterization of SMC

To determine the swelling ratio (*S*), the SMC sample was washed with water and weighed (*m*_wet_ gel). It was then dried to constant mass at 60 °C (*m*_dry_ gel) [37]. The swelling degree was calculated according to the following formula:

$$S=\frac{\left(m_{wet}-m_{dry}\right)}{m_{dry}}$$

Flow rate was determined by monitoring the volume of liquid eluted from the SMC column per unit time (mL/min). All measurements were performed in triplicate. The flow resistance of the affinity SMC column was determined using Darcy’s law, as described in the corresponding equation. The was determined according to the following equation [21]:

$$K=\frac{Q\cdot µ\cdot L}{A\cdot ∆P}$$

where *Q* is the volumetric flow rate of the fluid, *A* is the surface area of the SMC, ∆*P* is the differential pressure, *L* is the length of the column, and µ is the µ-fluid viscosity

The porosity of the synthesized SMC, both before and after VHH immobilization, was determined using mercury intrusion porosimetry (MIP). Prior to measurements, a dry cylindrical sample of appropriate mass was placed in a CD3 dilatometer. Low-pressure measurements (0.0136–0.1 MPa) were conducted using a Macropore Unit 120 (Carlo Erba Strumentazione), preceded by a 2 h drying step under vacuum (<1 kPa). This unit was also employed to introduce mercury into the dilatometer for determining sample pore volume in the range of 110 μm to 14.7 μm, as well as bulk density determination. To extend the pore diameter range coverage, a Porosimeter 2000 (Fisons Instruments, Glasgow, UK) was used for high-pressure measurements (0.1–200 MPa), covering pore diameters from 14.7 μm down to 7.5 nm. Data acquisition and calculation of key parameters—including porosity, total pore volume, bulk density (by Hg pycnometry), apparent density (by Hg porosimetry), mean, and most frequent pore diameter—were performed using the Milestone 200 interface and PASCAL ver. 1.05 software package, respectively.

For porosity determination of SMC and VHH-SMC, the true density of both samples was measured at (20.00 ± 0.01) °C using a Pycnomatic ATC gas pycnometer (Thermo Scientific, Thermo Scientific, Waltham, MA, USA) equipped with integrated automatic temperature control. Five measurement cycles were performed, with the true density value calculated as the mean of these cycles with a 95% confidence interval. Each cycle was repeated until five consecutive measurements showed a standard deviation difference of less than 0.1%. The porosity was calculated according to the equation.

$$P=1-\frac{\rho_{bulk}}{\rho_{t}}$$

where *ρ_bulk_* and *ρ_t_* are the bulk density determined by Hg pycnometry and true density determined by He pycnometry of the samples, respectively.

The morphology of the affinity SMC was examined using a field emission scanning electron microscope (Tescan (Brno, Czech Republic) FE-SEM Mira 3 XMU) operated at an accelerating voltage of 20 kV. Before imaging, the SMC samples were dehydrated in an oven at 60 °C for 48 h to remove residual moisture. A small section was then cut from the central region of the dried SMC and coated with a thin layer of gold using a sputter coater (Polaron SC503, Fisons Instruments) to improve conductivity and reduce surface charging during SEM analysis.

### 4.4. VHH Production

VHH antibodies (H1, H6, D5, B1, and G2) were isolated previously from a naïve pre-immune library using direct panning against extracellular vesicles (EVs) obtained from cell culture supernatants [15]. The VHH sequences were subcloned into a modified pET-14b vector between Nco I and Not I restriction sites to produce fusion proteins with C-terminal eGFP and 6×His tag [38]. The constructs were introduced into *Escherichia coli* BL21(DE3) cells containing plasmids for the production of sulfhydryl oxidase and DsbC isomerase [39,40,41]. The production of VHH antibodies adhered to a modified version of previously reported methods [14,15]. In summary, 1 mL of overnight pre-cultures were introduced into 400 mL of LB medium with 100 µg/mL ampicillin and 25 µg/mL chloramphenicol. Cultures were incubated at 37 °C until an OD600 of 0.4 was attained. The expression of DsbC and sulfhydryl oxidase was stimulated by the addition of 0.2% arabinose, and the temperature was lowered to 30 °C. After 30 min, IPTG (0.2%) was introduced to promote VHH expression, followed by overnight incubation at 21 °C. Cells were subsequently collected via centrifugation, and the pellet was reconstituted in 20 mL of TBS buffer (50 mM Tris-HCl, 500 mM NaCl, 5 mM MgCl_2_, pH 7.4). Following sonication, lysates were centrifuged at 12,000× *g* for 20 min at 4 °C, and the supernatant was subjected to immobilized metal affinity chromatography (IMAC). The resin was equilibrated with buffer A (50 mM Tris, 500 mM NaCl, 30 mM imidazole, pH 8), and unbound proteins were removed using the same buffer. Bound proteins were eluted using buffer B (50 mM Tris, 200 mM NaCl, 300 mM imidazole, pH 8). Fractions containing VHH-eGFP fusion proteins were pooled and 20% glycerol was included for prolonged preservation.

### 4.5. SMC Functionalization and Immobilization of VHH-eGFP on the SMC Column

**Figure 11:**
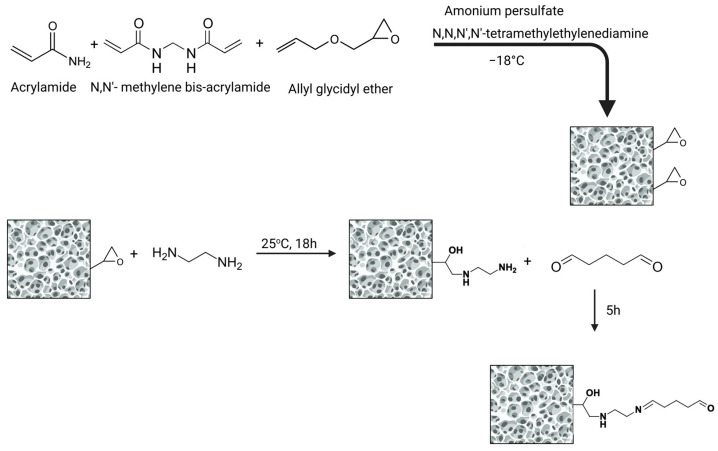
Schematic representation illustrating the activation of epoxy-functionalized cryogel for covalent immobilization of VHH nanoantibodies.

The AAm-AGE SMC was treated with ethylenediamine (1 M EDA in 0.2 M Na_2_CO_3_), which reacts with the epoxy groups and opens them. Afterward, the column was washed with water and 0.1 M sodium-phosphate buffer, pH 7, until a neutral pH was reached [42]. A solution of glutaraldehyde (5% *v*/*v*) in 0.1 M sodium-phosphate buffer, pH 7 was applied to the column for 4 h in recirculation mode (Figure 11). Subsequently, the excess glutaraldehyde was washed with 0.1 M sodium-phosphate buffer. VHH-eGFP was added to the column and allowed to circulate for 24 h.

### 4.6. Blood Collection and Plasma Isolation

Plasma samples were collected from healthy volunteers in compliance with the Declaration of Helsinki, following informed consent and approval by the Ethics Committee of the Faculty of Chemistry, University of Belgrade (2-6/24). Peripheral venous blood was drawn into citrate-containing Vacutainer tubes and processed within 30 min of collection. To remove blood cells, an initial centrifugation was performed at 1550× *g* for 30 min at room temperature, followed by a second centrifugation at 3200× *g* for 30 min to obtain platelet-free plasma (PFP). PFP aliquots were either used immediately for EV isolation or stored at −80 °C for future analysis.

### 4.7. Extracellular Vesicle Isolation from Human Plasma via SMC-Based Affinity Chromatography

The VHH-SMC was subjected to blocking with 200 mM glycine at pH 7 and 5% (*w*/*v*) milk to eliminate any residual surface groups. Subsequently, the sample was washed with 0.1 M sodium phosphate at pH 7.0. PFP (0.5 mL) was then applied, diluted 1:4 with 0.1 M sodium phosphate at pH 7.0, and recirculated for 2 h under a constant flow rate of 0.1 mL/min. Excess unbound proteins were removed using 0.1 M sodium phosphate at pH 7.0 until a negative reaction to proteins. Bound vesicles were eluted using glycine at pH 2.0 and subsequently collected in a tube containing 1 M Tris-HCl, pH 9.1, for pH neutralization. Individual 250 µL fractions were collected and analyzed by Bradford assay (595 nm) to monitor the elution profile. The sample was concentrated up to 1 mL. Reproducibility was evaluated by performing three consecutive isolations using the same cryogel column and the same plasma sample from a healthy donor.

### 4.8. Determination of EVs Proteins and Lipids

The protein concentration in EV isolates was determined with the BCA Protein Assay Kit (Abcam, Waltham, MA, USA) according to the manufacturer’s instructions using BSA as a standard. Lipid concentration was determined using standards of cholesterol (25–200 µg/mL) dissolved in chloroform. Aliquots (70 µL) of standards and samples were placed in microtubes, and the solvent was evaporated by incubation at 90 °C with open lids. Dried standards were resuspended in 50 µL of PBS, while 50 µL of vesicle suspension was added to the dried sample tubes. After incubation for 20 min at 90 °C, 250 µL of concentrated H_2_SO_4_ was added to each tube. The mixtures were transferred to a 96-well plate, with 220 µL of sample per well, and allowed to attain room temperature. Then, 110 µL of vanillin reagent (0.2 mg/mL in 17% H_3_PO_4_) was added, and the mixture was incubated for 10 min. The absorbance was determined at 540 nm. Lipid concentration was expressed as cholesterol equivalents calculated from the standard curve.

### 4.9. Flow Cytometry

Latex beads (3 µM, Sigma-Aldrich) were diluted in PBS to obtain a 1% suspension. A mixture of 4 µg of vesicle proteins and 30 µL of latex beads suspension was gently agitated and incubated at 4 °C overnight. The beads were blocked in two subsequent phases the following day: first, with 200 mM glycine for 30 min, and then with 5% skimmed milk in PBS for an additional 30 min. Following a thorough PBS wash to eliminate any remaining blocking agents, separate aliquots of commercial antibodies anti-CD9, anti-CD81, and anti-CD63 that target extracellular vesicle markers were added. Each aliquot was diluted 1:5 in 1% BSA, and the samples were incubated for 1 h at 37 °C. The antibody-coated beads were examined using a BD FACS Calibur system (BD Biosciences, Franklin Lakes, NJ, USA) that features a 488 nm blue laser. Emissions were measured at specific wavelengths for each antibody: 620 nm (PE/Dazzle) for anti-CD81, 525 nm (AlexaFluor 488) for anti-CD63, and 561 nm (PE) for anti-CD9. Milk-blocked, non-coated beads functioned as negative controls. Detergent controls have been performed by incubating samples with 0.5% Triton X-100 for 30 min at RT.

### 4.10. Nanoparticle Tracking Analysis (NTA)

The size and concentration of the EV samples were determined using the ZetaView Quatt PMX-430 nanoparticle tracking analyzer and ZetaView software version 8.05.16 SP3 (Particle Metrix, Inning am Ammersee, Germany). Before the analysis began, the camera and laser were automatically calibrated, and focus was verified using 100 nm polystyrene beads in accordance with the manufacturer’s guidelines. Measurements were performed using a blue laser at 488 nm in light scatter mode-LSM. Video recording was performed with a shutter speed of 100 and a frame rate of 30 frames per cycle, while the sensitivity was set to 78. Following the acquisition, the analysis parameters were set to a minimum particle area of 10, a maximum area of 1000, and a minimum brightness threshold of 30. EV samples were diluted in particle-free 0.05 M PBS (pH 7.2) to obtain an optimal number of particles per frame for analysis. Extracellular vesicle sample was analyzed in three measurements at up to 11 positions, to ensure data reliability.

### 4.11. Atomic Force Microscopy

Atomic force microscopy (AFM) was used to assess the surface morphology of the samples, employing a NanoScope 3D system (Veeco, Plainview, NY, USA). Etched silicon cantilevers with a spring constant ranging from 20 to 80 N/m were used for imaging. Before the sample application, the mica substrate was gently polished with adhesive tape. A few microliters of the extracellular vesicle suspension were deposited onto the prepared surface and allowed to air-dry. Image analysis was performed using Nanoscope software (version 1.40r1).

### 4.12. Scanning Electron Microscopy

Scanning electron microscopy (SEM) was employed to analyze the morphology of purified EVs. The EVs samples were deposited onto metal stubs and left to dry naturally in air. Before starting SEM analysis, samples were coated with a gold layer using a sputter coater (Polaron SC503, Fisons Instruments, Glasgow, Loughborough, UK). Morphological characterization was carried out using a Tescan Fe-SEM Mira 3XMU (Tescan, Brno, Czech Republic).

### 4.13. Protein Precipitation and Peptide Preparation for Mass Spectrometry

Suspended EVs were aliquoted in five technical replicates and processed in parallel. Precipitation of 4 µg of protein was performed by adding six volumes of cold acetone and incubating the mixture at 4 °C for 1 h. Samples were centrifuged at 15,000 rpm for 10 min at 4 °C, and the supernatant was carefully removed. The protein pellets were solubilized in 15 µL of LC-MS-grade trifluoroacetic acid (TFA) and vortexed. The acid was neutralized by adding 90 µL of 2 M Tris base. Subsequently, 10 µL of a freshly prepared 1:1 solution of tris(2-carboxyethyl) phosphine (TCEP, final concentration 10 mM) and chloroacetamide (CAA, 40 mM), along with 180 µL of Milli-Q water, were added. Samples were incubated at 95 °C for 5 min with agitation at 350 rpm, and then allowed to cool to room temperature. Proteins were digested by the addition of 40 ng of trypsin (2 μL trypsin working solution at 20 ng/µL in tris base 0.1 M). Digestion was performed overnight at 37 °C at 450 rpm. The next day, 5 µL of 100% TFA was added to each sample, and the pH was confirmed to be acidic. Peptides were desalted using C18 as previously described [43]. The stage tips were conditioned with 250 µL of acetonitrile and equilibrated with 250 µL of elution solvent (40% acetonitrile, 0.5% acetic acid), followed by 250 µL of solvent A (0.5% acetic acid in water), each step performed by centrifugation at 4000 rpm for 5 min at 25 °C. Acidified peptide samples were loaded on the C18 microcolumns, washed with 250 μL solvent A, and eluted with 40 µL of elution solvent. After drying overnight in a vacuum at 2000 rpm, room temperature, peptides were reconstituted in 40 µL of solvent A. The final peptide solutions were analyzed by mass spectrometry.

### 4.14. Mass Spectrometry

The method has been detailed elsewhere [43]. The peptides were analysed in a Bruker (Billerica, MA, USA) timsTOF fleX mass spectrometer operated in Data Independent Acquisition (DIA) mode. The resulting spectra were analysed with FragPipe v23 and DIA-NN v2.0 in a library-free mode [43]. Lists of identified proteins were obtained from the 5 technical replicates, with 62% of proteins quantified with a coefficient of variation below 20% across the five replicates, showing good repeatability of the proteomics analysis. Proteins identified in at least 2 replicates are listed in Supplementary Table S1, and their presence in respective replicates is displayed in Supplementary Figure S1 showing excellent overlap (Venn diagram created with https://bioinformatics.psb.ugent.be/webtools/Venn/, accessed on 2 October 2025).

### 4.15. STRING-Based Analysis of EV-Associated Proteins

Protein–protein interaction (PPI) analysis was performed using the STRING database (version 12.0; https://string-db.org) to examine the functional connectivity of the proteins identified in the extracellular vesicle samples. Only proteins previously confirmed as EV-specific by cross-checking with the publicly available ExoCarta (http://www.exocarta.org/) and Vesiclepedia databases (http://www.microvesicles.org/) were included in the analysis. Proteins known as common contaminants from plasma or serum, including albumin (ALB), apolipoproteins (APOA1, APOB, APOE), immunoglobulin chains (IGHG, IGKV, IGLV), and hemoglobin (HBA, HBB), as well as keratins (KRT1, KRT10, KRT14), were not included in the analysis. This provides an analysis that reflects a representative EV proteomic profile. The STRING network was generated using a medium confidence threshold (0.4), and evidence channels were included: curated databases, experimental evidence, text mining, and co-expression.

### 4.16. FunRich Analysis

Functional annotation of the identified EV-proteins was performed using the software tool FunRich (version 3.1.3). Only EV-specific proteins, confirmed by comparison with ExoCarta and Vesiclepedia databases, were included in the analysis. The analysis was focused on the Gene Ontology (GO) category Cellular Component, to identify the predominant subcellular localization of proteins. The statistical significance of the enrichment of GO terms is expressed by *p*-values corrected by the Benjamini–Hochberg method, and the results are shown as the percentage of proteins present in each category.

## 5. Conclusions

The cryogel-based immunoaffinity chromatography system functionalized with VHH single-domain antibodies described in this contribution successfully provided specific capture of human plasma-derived EVs. The supermacroporous cryogel porosity, flow characteristics, mechanical stability, and functionalization opportunity for VHHs allowed the rapid recovery of intact EVs, the identity of which was confirmed by microscopy, NTA, flow cytometry, and proteomic analyses that revealed good enrichment of exosome-associated proteins.

Relative to classical ultracentrifugation or polymer precipitation, the procedure is rapid and gentle, preserving EV integrity. Further repeats will be necessary to confirm the presented data that otherwise indicate elevated reproducibility. The system was tested only with healthy donor material, but it has been conceived as a platform to isolate EVs specific to disease states. This developmental step will be dependent on the availability of suitable VHH.

In summary, VHH-functionalized cryogels represent a promising and flexible tool for the enrichment of EVs, with potential applications in biomarker discovery, therapeutic monitoring, and diagnostics in clinics, as long as further optimization and standardization are achieved.

## Figures and Tables

**Table 1:** Structural and physical properties of the SMC.

Swelling Ratio	Flow Rate	Hydraulic Permeability
16 g/g H_2_O	8 mL/min	1.296 × 10^−10^ m^2^

**Table 2:** MIP results of SMC before and after VHH functionalization.

Parameter	SMC	VHH-SMC
Total cumulative volume (V*_tot_*), cm^3^/g	3.00	6.53
Bulk density (*ρ_bulk_*), g/cm^3^	0.266	0.139
Apparent density (*ρ_App_*), g/cm^3^	1.31	1.48
True density (*ρ_t_*), g/cm^3^	1.351	1.519
entry 5		
Average pore diameter (D*_avg_*), µm	38.40	56.35
Most frequent pore diameter (D*_max_*), µm	29.46	41.10
Pore diameter at 90% of V*_tot_*, µm	24.84	41.75
Porosity (P), %	80.3	90.8

**Table 3:** Characteristics of EVs isolated from human plasma using VHH-functionalized SMC. Reported values are calculated per starting volume of plasma (0.5 mL).

Parameter	
Starting volume, mL	0.5
Protein yield, mg	1.21 ± 0.15
Lipid yield, mg	6.80 ± 0.34
Tetraspanin detection	CD9+/CD63+/CD81+
SEM	Single vesicles, 50–150 nm
AFM	Single vesicles, 50–150 nm
Particle yield × 10^9^	1.9 ± 0.1
Mean particle size, nm	135.6 ± 1.19

**Table 4:** L/P ratio of EVs obtained from three independent isolations.

Isolate	L/P Ratio
Isolate 1	5.56 ± 0.13
Isolate 2	6.37 ± 0.16
Isolate 3	5.12 ± 0.16

**Table 5:** Functional classification of EV-associated proteins identified in samples.

Functional Category	Protein
Cytoskeleton/structural proteins	ACTG1, DSP, JUP
ECM/adhesion	FN1, HSPG2, LGALS3BP, THBS1, VTN
Immune response/inflammation	B2M, CD14, CD5L, FCN3, LRG1, LYZ, MST1, PPBP, S100A7, S100A8, S100A9
Complement and coagulation	C1RL, C3, C4B, C4BPA, C4BPB, CFP, F2
Metabolism/stress response	GAPDH, GPX3, HSPA5, HSPB1, PKM, PON1, PRDX2
Transport/vesicle-related/others	AFM, CLU, CP, CTSD, DCD, GSN, HP, HPX, LCN2, ORM2, PC, PIP

## Data Availability

The data presented in this study are available on request from the corresponding author.

## Supplementary Materials

The following supporting information can be downloaded at: https://www.mdpi.com/article/10.3390/molecules30224337/s1, Figure S1: Venn diagram illustrating the overlap in identified proteins between the five EV samples; Figure S2: Integral and differential pore size distribution of SMC and VHH-SMC determined by mercury intrusion porosimetry; Table S1: Proteomic dataset of EV samples including calculated coefficients of variation (CVs).

## Abbreviations

The following abbreviations are used in this manuscript:

AAm	Acrylamide
AGE	Allylglycidyl Ether
EVs	Extracellular Vesicles
MBA	N,N′-Methylenebisacrylamide
NTA	Nanoparticle Tracking Analysis
SEC	Size-Exclusion Chromatography
SMC	Supermacroporous Cryogel
VHH	Single-Domain Antibodies/Nanobodies
VHH-SMC	Functionalized Supermacroporous Cryogel

## References

[1] Phillips W., Willms E., Hill A.F. (2021). Understanding extracellular vesicle and nanoparticle heterogeneity: Novel methods and considerations. Proteomics.

[2] Welsh J.A., Goberdhan D.C.I., O’Driscoll L., Buzas E.I., Blenkiron C., Bussolati B., Cai H., Di Vizio D., Driedonks T.A.P., Erdbrügger U. (2024). Minimal information for studies of extracellular vesicles (MISEV2023): From basic to advanced approaches. J. Extracell. Vesicle.

[3] Skotland T., Sagini K., Sandvig K., Llorente A. (2020). An emerging focus on lipids in extracellular vesicles. Adv. Drug Deliv. Rev..

[4] Matsuzaka Y., Yashiro R. (2022). Extracellular Vesicles as Novel Drug-Delivery Systems through Intracellular Communications. Membranes.

[5] Yokoi A., Ochiya T. (2021). Exosomes and extracellular vesicles: Rethinking the essential values in cancer biology. Semin. Cancer Biol..

[6] Kumar M.A., Baba S.K., Sadida H.Q., Marzooqi S.A., Jerobin J., Altemani F.H., Algehainy N., Alanazi M.A., Abou-Samra A.-B., Kumar R. (2024). Extracellular vesicles as tools and targets in therapy for diseases. Sig. Transduct. Target. Ther..

[7] Zeng Y., Qiu Y., Jiang W., Shen J., Yao X., He X., Li L., Fu B., Liu X. (2022). Biological Features of Extracellular Vesicles and Challenges. Front. Cell Dev. Biol..

[8] Isaac R., Reis F.C.G., Ying W., Olefsky J.M. (2021). Exosomes as mediators of intercellular crosstalk in metabolism. Cell Metab..

[9] Zarà M., Guidetti G.F., Camera M., Canobbio I., Amadio P., Torti M., Tremoli E., Barbieri S.S. (2019). Biology and Role of Extracellular Vesicles (EVs) in the Pathogenesis of Thrombosis. Int. J. Mol. Sci..

[10] Gandham S., Su X., Wood J., Nocera A.L., Alli S.C., Milane L., Zimmerman A., Amiji M., Ivanov A.R. (2020). Technologies and Standardization in Research on Extracellular Vesicles. Trends Biotechnol..

[11] Nieuwland R., Siljander P.R.-M., Falcón-Pérez J.M., Witwer K.W. (2022). Reproducibility of extracellular vesicle research. Eur. J. Cell Biol..

[12] Taylor D.D., Shah S. (2015). Methods of isolating extracellular vesicles impact down-stream analyses of their cargoes. Methods.

[13] Nakai W., Yoshida T., Diez D., Miyatake Y., Nishibu T., Imawaka N., Naruse K., Sadamura Y., Hanayama R. (2016). A novel affinity-based method for the isolation of highly purified extracellular vesicles. Sci. Rep..

[14] Filipović L., Spasojević M., Prodanović R., Korać A., Matijaševic S., Brajušković G., De Marco A., Popović M. (2022). Affinity-based isolation of extracellular vesicles by means of single-domain antibodies bound to macroporous methacrylate-based copolymer. New Biotechnol..

[15] Popovic M., Mazzega E., Toffoletto B., De Marco A. (2018). Isolation of anti-extra-cellular vesicle single-domain antibodies by direct panning on vesicle-enriched fractions. Microb. Cell Fact..

[16] Filipović L., Spasojević Savković M., Prodanović R., Matijašević Joković S., Stevanović S., Marco A.D., Kosanović M., Brajušković G., Popović M. (2024). Urinary Extracellular Vesicles as a Readily Available Biomarker Source: A Simplified Stratification Method. Int. J. Mol. Sci..

[17] Yao K., Shen S., Yun J., Wang L., He X., Yu X. (2006). Preparation of polyacrylamide-based supermacroporous monolithic cryogel beds under freezing-temperature variation conditions. Chem. Eng. Sci..

[18] He X., Yao K., Shen S., Yun J. (2007). Freezing characteristics of acrylamide-based aqueous solution used for the preparation of supermacroporous cryogels via cryo-copolymerization. Chem. Eng. Sci..

[19] Lozinsky V.I., Galaev I.Y., Plieva F.M., Savina I.N., Jungvid H., Mattiasson B. (2003). Polymeric cryogels as promising materials of biotechnological interest. Trends Biotechnol..

[20] Bakhshpour M., Idil N., Perçin I., Denizli A. (2019). Biomedical Applications of Polymeric Cryogels. Appl. Sci..

[21] Carvalho B.M.A., Da Silva S.L., Da Silva L.H.M., Minim V.P.R., Da Silva M.C.H., Carvalho L.M., Minim L.A. (2014). Cryogel Poly(acrylamide): Synthesis, Structure and Applications. Sep. Purif. Rev..

[22] Plieva F.M., Kirsebom H., Mattiasson B. (2011). Preparation of macroporous cryostructurated gel monoliths, their characterization and main applications. J. Sep. Sci..

[23] Okay O. (2023). Cryogelation reactions and cryogels: Principles and challenges. Turk. J. Chem..

[24] Lozinsky V.I. (2002). Cryogels on the Basis of Natural and Synthetic Polymers: Preparation, Properties and Applications. ChemInform.

[25] Ribeiro J., Luís M.Â., Rodrigues B., Santos F.M., Mesquita J., Boto R., Tomaz C.T. (2024). Cryogels and Monoliths: Promising Tools for Chromatographic Purification of Nucleic Acids. Gels.

[26] Arvidsson P., Plieva F.M., Lozinsky V.I., Galaev I.Y., Mattiasson B. (2003). Direct chromatographic capture of enzyme from crude homogenate using immobilized metal affinity chromatography on a continuous supermacroporous adsorbent. J. Chromatogr. A.

[27] Kumar A., Galaev I.Y., Mattiasson B. (2007). Cell Separation.

[28] Buzas E.I. (2023). The roles of extracellular vesicles in the immune system. Nat. Rev. Immunol..

[29] Margolis L., Sadovsky Y. (2019). The biology of extracellular vesicles: The known unknowns. PLoS Biol..

[30] Gill S., Catchpole R., Forterre P. (2019). Extracellular membrane vesicles in the three domains of life and beyond. FEMS Microbiol. Rev..

[31] Yáñez-Mó M., Siljander P.R.M., Andreu Z., Bedina Zavec A., Borràs F.E., Buzas E.I., Buzas K., Casal E., Cappello F., Carvalho J. (2015). Biological properties of extracellular vesicles and their physiological functions. J. Extracell. Vesicle.

[32] Suresh P.S., Zhang Q. (2025). Comprehensive Comparison of Methods for Isolation of Extracellular Vesicles from Human Plasma. J. Proteome Res..

[33] Shami-shah A., Travis B.G., Walt D.R. (2023). Advances in extracellular vesicle isolation methods: A path towards cell-type specific EV isolation. Extracell. Vesicles Circ. Nucleic Acids.

[34] Jia Y., Yu L., Ma T., Xu W., Qian H., Sun Y., Shi H. (2022). Small extracellular vesicles isolation and separation: Current techniques, pending questions and clinical applications. Theranostics.

[35] Wu Q., Fu S., Xiao H., Du J., Cheng F., Wan S., Zhu H., Li D., Peng F., Ding X. (2023). Advances in Extracellular Vesicle Nanotechnology for Precision Theranostics. Adv. Sci..

[36] Nambiar D., Le Q.-T., Pucci F. (2024). A case for the study of native extracellular vesicles. Front. Oncol..

[37] Demiryas N., Tüzmen N., Galaev I.Y., Pişkin E., Denizli A. (2007). Poly(acrylamide-allyl glycidyl ether) cryogel as a novel stationary phase in dye-affinity chromatography. J. Appl. Polym. Sci..

[38] Djender S., Beugnet A., Schneider A., De Marco A. (2014). The Biotechnological Applications of Recombinant Single-Domain Antibodies are Optimized by the C-Terminal Fusion to the EPEA Sequence (C Tag). Antibodies.

[39] Nguyen V.D., Hatahet F., Salo K.E., Enlund E., Zhang C., Ruddock L.W. (2011). Pre-expression of a sulfhydryl oxidase significantly increases the yields of eukaryotic disulfide bond containing proteins expressed in the cytoplasm of *E. coli*. Microb. Cell Factories.

[40] Veggiani G., De Marco A. (2011). Improved quantitative and qualitative production of single-domain intrabodies mediated by the co-expression of Erv1p sulfhydryl oxidase. Protein Expr. Purif..

[41] Crépin R., Gentien D., Duché A., Rapinat A., Reyes C., Némati F., Massonnet G., Decaudin D., Djender S., Moutel S. (2017). Nanobodies against surface biomarkers enable the analysis of tumor genetic heterogeneity in uveal melanoma patient-derived xenografts. Pigment Cell Melanoma Res..

[42] Ingavle G.C., Baillie L.W.J., Zheng Y., Lis E.K., Savina I.N., Howell C.A., Mikhalovsky S.V., Sandeman S.R. (2015). Affinity binding of antibodies to supermacroporous cryogel adsorbents with immobilized protein A for removal of anthrax toxin protective antigen. Biomaterials.

[43] Karousi P., Voumvouraki M., Nikolaou P.E., Kollias I., Paradeisi F., Sampanai E., Gkalea V., Morianos I., Zoidakis J., Kastritis E. (2025). The privilege of a new core facility: Optimising technical repeatability and workflow efficiency in proteomics across diverse biological matrices. Authorea.

