# Supplementary material for: Application of VHH-Immobilized Cryogel-Based Immunoaffinity Chromatography for Isolation of Extracellular Vesicles
Source: Molecules. 2025 Nov 8;30(22):4337. doi: 10.3390/molecules30224337 (PMC12654627; doi:10.3390/molecules30224337)

Figure S1: Venn diagram illustrating the overlap in identified proteins between the 5 samples of EVs

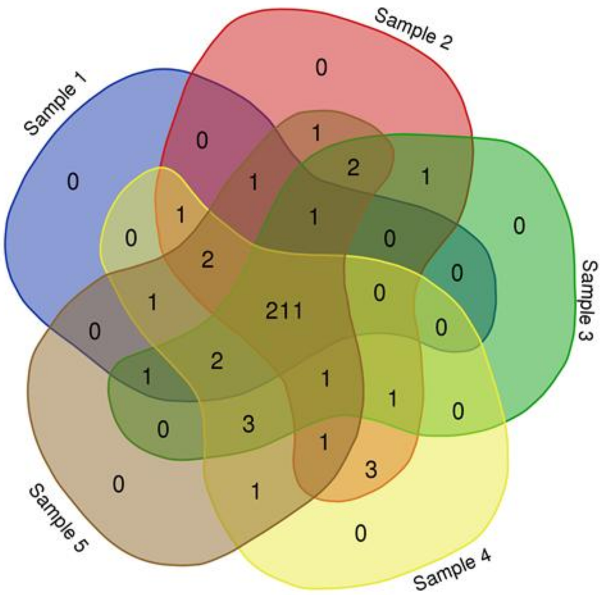

Figure S2. Integral and differential pore distribution of SMC and VHH-SMC determined by Hg intrusion porosimetry

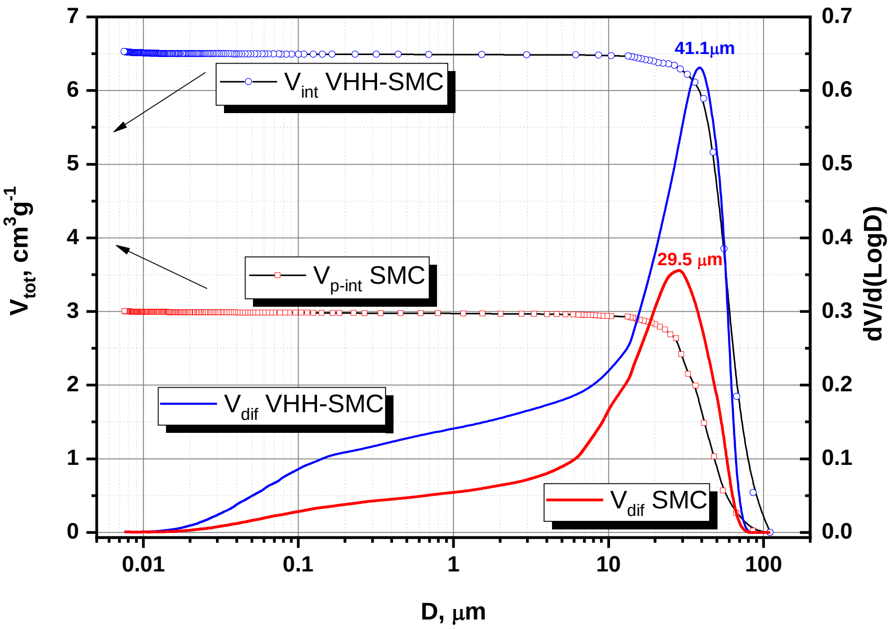

Supplement: Supplementary file 1 [file molecules-30-04337-s001.zip › molecules-3955009-Supplementary Figures.pdf]
